# Amygdala activation and GABAergic gene expression in hippocampal sub-regions at the interplay of stress and spatial learning

**DOI:** 10.3389/fnbeh.2014.00003

**Published:** 2014-01-21

**Authors:** Osnat Hadad-Ophir, Anne Albrecht, Oliver Stork, Gal Richter-Levin

**Affiliations:** ^1^Sagol Department of Neurobiology, University of HaifaHaifa, Israel; ^2^The Brain and Behavior Research Center, University of HaifaHaifa, Israel; ^3^The Institute for the Study of Affective Neuroscience, University of HaifaHaifa, Israel; ^4^Department of Genetics and Molecular Neurobiology, Institute of Biology, Otto-von-Guericke University MagdeburgGermany; ^5^Center for Behavioral Brain SciencesMagdeburg, Germany

**Keywords:** basolateral amygdala, hippocampus, Morris water maze (MWM), stress, interneuron, gene expression, rat

## Abstract

Molecular processes in GABAergic local circuit neurons critically contribute to information processing in the hippocampus and to stress-induced activation of the amygdala. In the current study, we determined expression changes in GABA-related factors induced in subregions of the dorsal hippocampus as well as in the BLA of rats 5 h after spatial learning in a Morris water maze (MWM), using laser microdissection and quantitative real-time PCR. Spatial learning resulted in highly selective pattern of changes in hippocampal subregions: gene expression levels of neuropeptide Y (NPY) were reduced in the hilus of the dentate gyrus (DG), whereas somatostatin (SST) was increased in the stratum oriens (SO) of CA3. The GABA-synthesizing enzymes *GAD65* and *GAD67* as well as the neuropeptide cholecystokinin (CCK) were reduced in SO of CA1. In the BLA, expression of *GAD65* and *GAD67* were reduced compared to a handled Control group. These expression patterns were further compared to alterations in a group of rats that have been exposed to the water maze but were not provided with an invisible escape platform. In this Water Exposure group, no expression changes were observed in any of the hippocampal subregions, but a differential regulation of all selected target genes was evident in the BLA. These findings suggest that expression changes of GABAergic factors in the hippocampus are associated with spatial learning, while additional stress effects modulate expression alterations in the BLA. Indeed, while in both experimental groups plasma corticosterone (CORT) levels were enhanced, only Water Exposure stress activated the basolateral amygdala (BLA), as indicated by increased levels of phosphorylated ERK 1/2. Altered GABAergic function in the BLA may thus contribute to memory consolidation in the hippocampus, in relation to levels of stress and emotionality associated with the experience.

## Introduction

Information processing in the hippocampus depends on a fined tune interaction of excitatory and inhibitory systems (Ego-Stengel and Wilson, 2007). Inhibitory neurotransmission is defined by the action of γ-aminobutyric acid (GABA) that is synthesized via two isoenzymes, glutamic acid decarboxylase (GAD)65 and GAD67, in so-called interneurons. While pharmacological studies using classical modulators of GABA receptors indicate a rather memory-impairing role of GABAergic neurotransmission in hippocampus-dependent tasks (D'Hooge and De Deyn, 2001; Myhrer, 2003), more recently developed drugs, e.g., the GABA analog Gabapentin (Celikyurt et al., 2011), show the reverse effect. The involvement of GABAergic neurotransmission in hippocampus-dependent tasks therefore appears complex and requires a careful evaluation to exclude potentially confounding side effects of drugs. Moreover, GABA is expressed in diverse populations of interneurons with different functions across hippocampal subregions (Freund and Buzsaki, 1996). These subpopulations are characterized by specific expression of calcium-binding proteins and neuropeptides like neuropeptide Y (NPY), somatostatin (SST) and also cholecystokinin (CCK). These neuropeptides themselves modulate inhibition and excitation in local circuits (Sperk et al., 2007; Tallent, 2007; Lee and Soltesz, 2011) and affect thereby also hippocampal tasks as spatial learning in a Morris water maze (MWM) (Thorsell et al., 2000; Dyer and Cain, 2007; Lo et al., 2008).

We previously demonstrated that spatial learning in a MWM under different levels of stress alters synaptic plasticity in the hippocampus in a region specific-manner (Kavushansky et al., 2006). Such dissociated responses within the hippocampal subregions cornu ammonis field 1 (CA1) and the dentate gyrus (DG) have been observed particularly after activation of the basolateral amygdala (BLA) complex (Vouimba et al., 2004; Vouimba and Richter-Levin, 2005). Amygdala inputs appear to regulate the expression of different interneuron subpopulation markers in distinct subregions of the hippocampus (Berretta et al., 2001, 2004) and exposure to stress alters GABAergic functioning at the molecular and physiological levels in the hippocampus (Jacobson-Pick et al., 2008; Yarom et al., 2008; Jacobson-Pick and Richter-Levin, 2010). GABAergic signaling within the BLA is also modulated by stress (e.g., Bergado-Acosta et al., 2008; Heldt et al., 2012) and such changes have the potential to modulate synaptic plasticity within the hippocampus as well as hippocampus-dependent spatial memory (Kim et al., 2005). These findings suggest an involvement of the GABAergic system in both the amygdala and hippocampus during spatial learning and a modulation of their interaction under conditions of elevated stress.

In this study we began to address this question by investigating gene expression of GABAergic factors, including the key enzymes for GABA synthesis, *GAD65* and *GAD67*, and GABA-associated neuropeptides in subregions of the hippocampus and in the BLA after a spatial learning task in the MWM. MessengerRNA (mRNA) levels were compared to a group of animals that experienced comparable physical stress exposure to the water without an invisible platform to locate. The increased stress response due to the water maze experience was confirmed in both groups by assessing corticosterone (CORT) plasma levels. Through measurement of ERK 1/2 phosphorylation, we demonstrated a differential activation of the amygdala in rats that have acquired the spatial learning task compared to animals that have been water exposed only. The differential regulation of mRNA expression levels of selected target genes under these conditions provide evidence for a differential, stress- and memory related regulation of GABA interneuron function in different hippocampal subregions and within the amygdalo-hippocampal system.

## Methods

### Animals

The experiments were carried out in accordance with the guidelines of the University of Haifa Ethics and Animal Care Committee. Male Sprague–Dawley rats were obtained at an age of 8–10 weeks (weight 200–300 g) from Harlan Laboratory (Jerusalem, Israel). Animals were maintained in groups of 4 on a 12 h light: 12 h dark cycle (lights on 07.00 am) with food and water *ad libitum*. After 5 days of acclimation rats were assigned to behavioral training.

### Behavioral training

Rats trained in the water maze [(Morris, 1984), (diameter: 1.70 m; rim high: 50 cm; water temperature 23 ± 1°C)], were randomly assigned to three groups: The Spatial Learning group, trained to locate a hidden platform (12 × 12 cm, fixed location 30 cm away from rim, 1.5–2 cm beneath the water surface) in 12 trials 4 min inter trial intervals; adapted from (Akirav et al., 2001). After 60 s animals that failed to reach the platform were guided by the experimenter. Videos were recorded for each trial and escape latencies were measured using a stopwatch. The water exposure stress group underwent yoked training, i.e., no platform was placed in the water maze, but these animals underwent the same number of trials with matched exposure time to the water as learning curve of the Spatial Learning group (Figure S1). An additional Control group was handled once a day for three consecutive days but was not exposed to swim stress.

### Brain preparation

Brain tissue was processed for two different types of analysis. One batch (*N* = 8 Spatial Learning; *N* = 8 Water Exposure; *N* = 8 Control) was decapitated 5–10 min after the last water maze trial and trunk blood was collected. The BLA was manually dissected on 1 mm thick slices with sterile razor blades, leaving out the central amygdala (CeA). BLA samples were immediately frozen in liquid nitrogen and stored at −80°C until further analysis.

Another batch of rats (*N* = 9 for each group) was deeply anaesthetized 5 h after the last water maze trial by chloral hydrate i.p injection (15 mg/kg) and perfused transcardially with 100 ml ice-cold Tyrode Buffer containing 0.02% heparine sodiam sulfate (25000I.E.; Braun Melsung, Melsung, Germany), followed by 300 ml of cold 4% paraformaldehyde (PFA) in 0.1 M phosphate buffer. Brains were rapidly removed, postfixed in the same fixative for 24 h at 4°C and immersed for 24 h in 30% sucrose solutions (Sigma-Aldrich, Seelze, Germany) with sodium azide 0.02% (Riedel–de Haen, Seelze, Germany) for cryo protection. Brains were snap frozen in liquid nitrogen-cooled methylbutane and stored at −80°C until laser capture microdissection of areas of interest took place.

### Cort radioimmunoassay

Trunk blood samples were centrifuged at 3500 r.p.m. for 10 min at 4°C. ~500 μl serum of each animal were gained and stored at −20°C. CORT plasma levels were assessed using DSL/10/81000 ELISA kit (DSL, Texas).

### p-ERK 1/2 western blotting

Frozen BLA samples were homogenized in 300 μl Urea lysis buffer (1 mM EDTA, 0.5% Triton-X, 6 M urea, 100 μM PMSF) with freshly added protease and phosphotase inhibitors (0.1 mM sodium orthovanadate, 1 lg/ml leupeptine, 1.6 lg/ml aprotinin, 5 mM NaF, and 1 lg/ml protease inhibitor cocktail P2714; Sigma, Rehovot, Israel) and incubated at 100°C for 5 min. 10 μg samples were loaded on 10% SDS-polyacrylamide gel for electrophoresis (SDS-PAGE). After semi-dry transfer (nitrocellulose membrane) and blocking of unspecific bindings, incubation with primary antibodies took place (over night at 4°C): α-ERK 1/2 (p44/42 MAP kinase) and α-p-ERK 1/2 (phospho-p44/42 MAP kinase; Thr202/Tyr204; Cell Signaling, Beverly, MA; 1:1000); followed by secondary α-rabbit antibody (polyclonal; 1:10000) incubation and chemiluminescence detection. Using Quantity One 1-D Analysis software, ratios between the phosphorylated and the non-phosphorylated form of ERK 1/2 were calculated for each sample and normalized to the average of the Control group.

### LCM and quantitative real-time PCR

Gene expression was assessed using laser capture microdissected (LCM) for collecting subregions of the hippocampus and in the BLA. 20 μm cryosections were cut at the level of amygdala and dorsal hippocampus from PFA-fixed brains, thaw mounted on the PLL-coated (0.05% Poly-L-Lysine) RNase free membrane slides and allowed to dry on a warming plate at 40°C to minimize RNase activity. Sections were fixed with 70% ethanol (1 min at −20°C) and stained with 1% Cresyl Violet acetate solution (50% ethanol/DMDC-treated Aqua dd.; 1 min at 4°C). After dehydration in an increasing ethanol series (70–96% ethanol, in DMDC-Aqua dd.; 2 min at 4°C each) sections were air-dried and LCM took place immediately after. The hilus of the dorsal DG and the stratum oriens (SO) of the dorsal CA1 and 3 in the hippocampus as well as BLA were identified in a 10-fold magnification under the microscope of the LCM setup (Carl Zeiss, Jena, Germany) and marked at the digital life image on the computer screen. Target regions were sampled from the left and right hemisphere of 20–24 sections per animal and collected in a capture device (CloseCut and AutoLPC mode with 70% Energy).

Sample lysis and subsequent isolation of total RNA via a spin column system was conducted with the RNeasy FFPEKit, (QIAGEN, Hilden, Germany) according to manufacturer's instructions, including steps for removal of genomic DNA. Extraction of RNA failed in some samples (in that case, *N* = 8). RNA samples were stored at −80°C until further processing.

First-strand synthesis of cDNA was performed with the Sensiscript Reverse Transcription kit (QIAGEN, Hilden, Germany), specifically designed for low amounts of RNA, in the presence of 2.5 mM dNTPs as well as 50 μM random decamer first strand primers and RNase Inhibitor (SuperaseIN; 20 U/μl; both Life Technologies, Darmstadt, Germany) at 42°C for 60 min followed by enzyme inactivation at 94°C for 10 min. A 1:5 dilution of cDNA samples was used for determination of expression levels of selected target genes by quantitative PCR using the ABI Prism Step One real time PCR apparatus (Life Technologies, Darmstadt, Germany) and TaqMan^®^ reagents with predesigned assays for *GAD65* (*Gad2*; assay ID Rn00561244_m1), *GAD67* (*Gad1*; assay ID Rn00566593_m1), *NPY* (assay ID Rn00561681_m1), *SST* (assay ID Rn00561967_m1), *CCK* (assay ID Rn00563215_m1) and the housekeeping gene glycerinaldehyd-3-phosphat-dehydrogenase (*GAPDH*; endogenous control, Life Technologies, Darmstadt, Germany). Target and housekeeping genes were labeled with different fluorescent dyes, allowing for quantitative multiplex PCR. Samples were run in triplicate assays, consisting of 50 cycles of 15 s at 95°C and 1 min at 60°C, preceded by a 2 min decontamination step at 50°C with Uracil-N-Glycosidase and initial denaturation at 95°C for 10 min.

For data analysis, the mean cycle threshold (CT) was determined for each triplicate assay and relative quantification of each target gene was conducted with the ddCT method (Livak and Schmittgen, 2001), normalizing each sample to the overall content of cDNA using *GAPDH* as an internal control {dCT; dCT = [CT (target gene)] − [CT (GAPDH)]}. Normalization of all ddCT values was done relative to Control group with ddCT = dCT(sample) − mean dCT (Control group). Transformation to RQ values for a specific target gene and area was done according to RQ = 2^−ddCT^ with RQ_(Control)_ = 1.

### Statistical analysis

One-Way ANOVA for the factor training group was conducted followed by LSD tests for *post-hoc* comparison. When the One-Way ANOVA could not be used because of normality problems, nonparametric test was conducted followed by Mann–Whitney U test.

## Results

### Gene expression in the hippocampus

**Figure 1:**
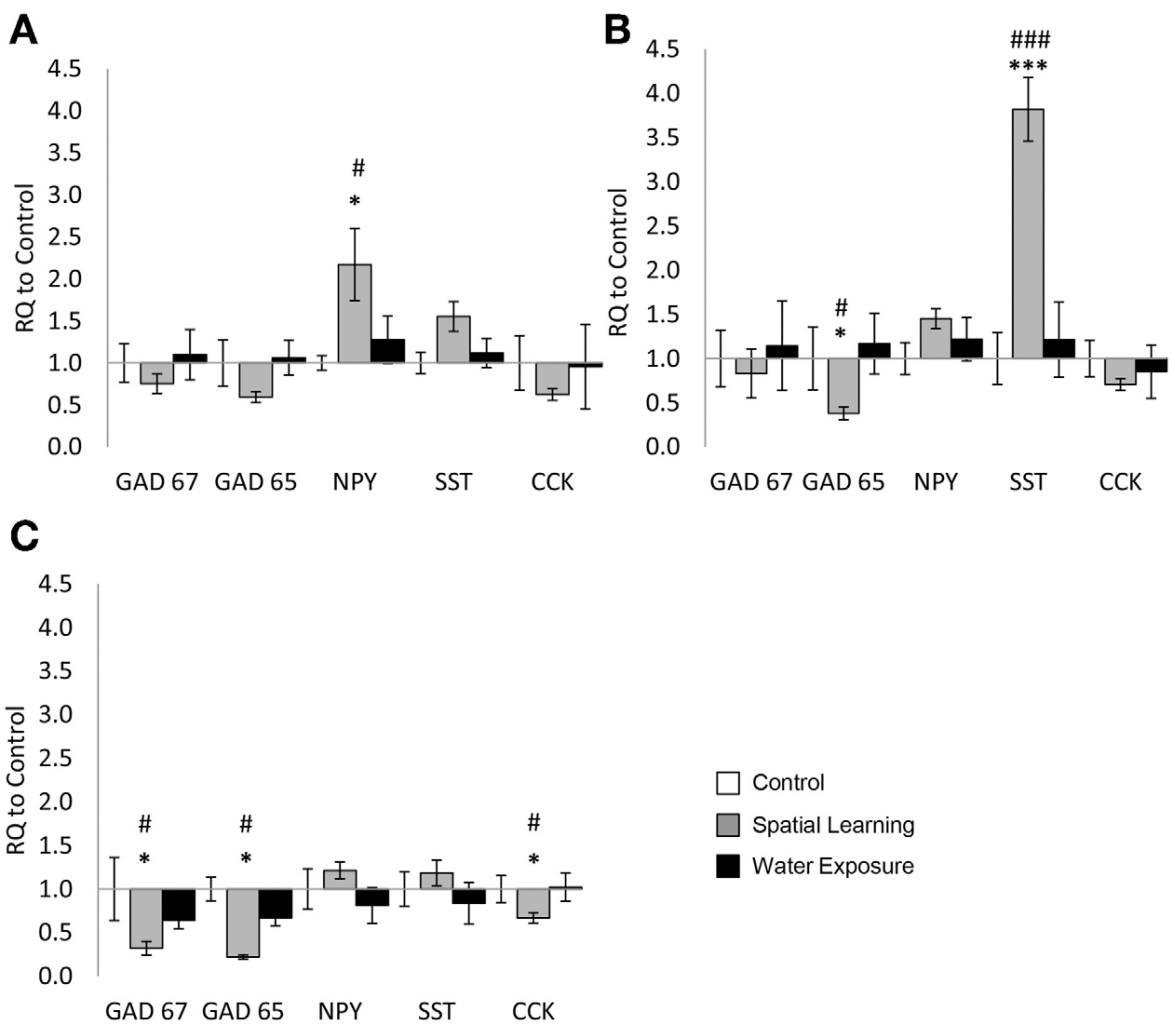
**Highly selective change of GABAergic gene expression in the hippocampus**. Differential mRNA expression changes of the selected GABAergic marker genes *GAD65* and *GAD67* as well as the neuropeptides neuropeptide Y (*NPY*), somatostatin (*SST*), and cholecystokinin (*CCK*) were observed in distinct hippocampal subregions 5 h after spatial learning utilizing an invisible platform vs. water exposure only. **(A)** In the hilus of the dentate gyrus *NPY* mRNA expression levels were increased in the Spatial Learning group. No significant differences between the groups were observed in other genes. **(B)** In the *stratum oriens* of the CA3 subregion *GAD65* mRNA levels were decreased as well in the Spatial Learning group, while SST expression was increased. **(C)** In the *stratum oriens* of the CA1 subregion, *GAD65*, *GAD67*, and *CCK* mRNA expression levels were decreased in the Spatial Learning group. Values are shown as relative quantification to handled controls [RQ; RQ_(Control)_ = 1] and mean ± s.e.m. per group. ^*^Significant difference from Control with *p* < 0.05; ^***^p < 0.001; ^#^significant difference between Spatial Learning and Water Exposure groups with *p* < 0.05; ^###^*p* < 0.001.

Expression of *GAD65*, *GAD67*, *NPY*, *SST*, and *CCK* was assessed in subareas of the hippocampus, revealing a highly region-specific pattern of changes. In the hilus, only expression of *NPY* differed significantly between groups [Figure 1A; One-Way ANOVA: *NPY*: *F*_(2, 23)_ = 4.377, *p* < 0.05; *GAD67*: *F*_(2, 23)_ = 0.896, n.s; *GAD65*: *F*_(2, 23)_ = 3.233, n.s; *CCK*: *F*_(2, 23)_ = 1.666, n.s]. LSD test for *post-hoc* comparison showed higher levels of *NPY* mRNA after exposure to the MWM with the escape platform (Spatial Learning group) compared to the Control group (*p* < 0.05), while increase compared to Water Exposure group failed to reach significant level (*p* = 0.055).

In the SO of the CA3 subregion only mRNA expression of *GAD65* and *SST* were significantly affected by MWM exposure [Figure 1B; *GAD65*: *F*_(2, 23)_ = 6.766, *p* < 0.01; *SST*: *F*_(2, 24)_ = 11.733, *p* < 0.001], with reduced *GAD65* expression after spatial learning, but significantly higher expression of *SST* in the same group (*p* < 0.001 from Water Exposure and Control group). No significant difference were observed for the other genes [Figure 1B; *GAD67*: *F*_(2, 23)_ = 0.468, n.s; *NPY*: *F*_(2, 24)_ = 1.396, n.s; *CCK*: *F*_(2, 24)_ = 0.830, n.s].

In the SO of the CA1 region finally, significant differences after MWM exposure were observed in the mRNA expression levels of *GAD65*, *GAD67*, and *CCK* [Figure 1C: One-Way ANOVA: *GAD67*: *F*_(2, 24)_ = 7.691, *p* < 0.05; *GAD65*: *F*_(2, 23)_ = 13.860, *p* < 0.01; *CCK*: *F*_(2, 24)_ = 3.715, *p* < 0.05]. For those, mRNA expression was reduced in the Spatial Learning group compared to Control and to the Water Exposure group (LSD *post-hoc* test: *p* < 0.05). No significant effect between the groups were observed for *SST* and *NPY* expression [Figure 1C: *SST*: *F*_(2, 24)_ = 1.024, n.s; *NPY*: *F*_(2, 23)_ = 1.148, n.s].

### Gene expression in the BLA

**Figure 2:**
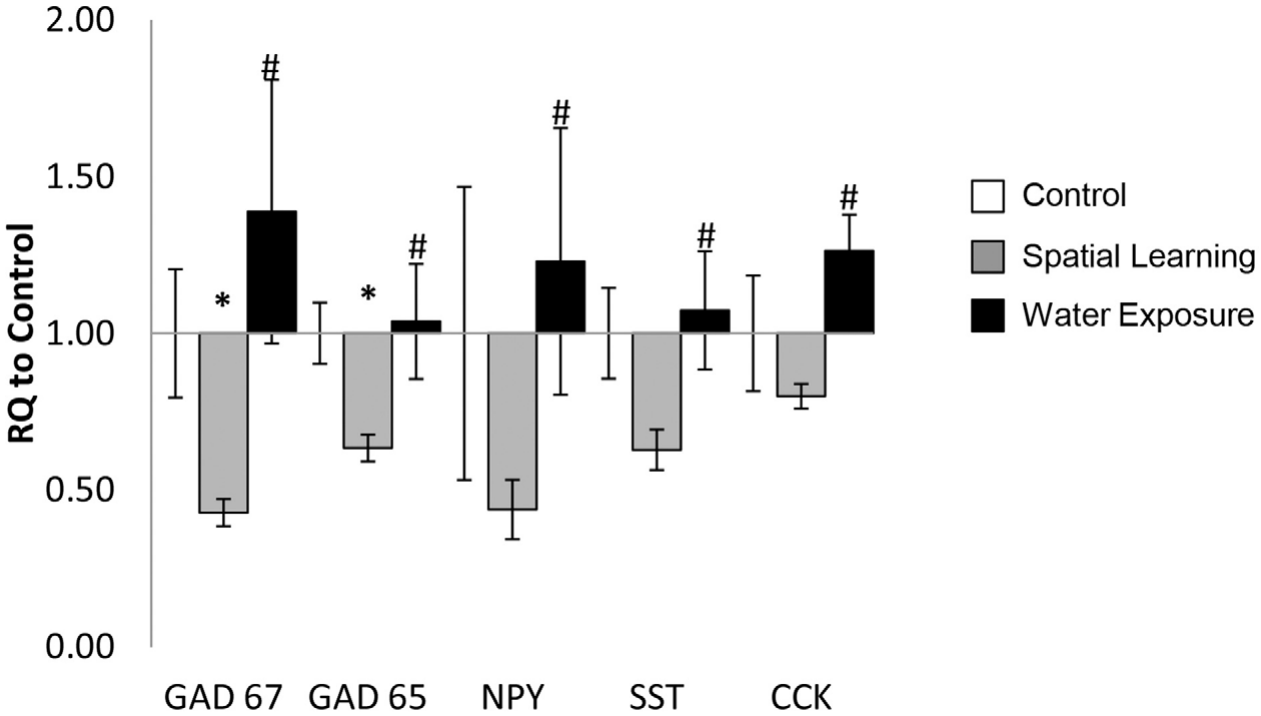
**Stress-specific change of GABA-related gene expression in the BLA**. The mRNA expression levels of the selected GABAergic marker genes *GAD65* and *GAD67* as well as the neuropeptides neuropeptide Y (*NPY*), somatostatin (*SST*), and cholecystokinin (*CCK*) were decreased in the in BLA 5 h after spatial learning in the water maze, but not after exposure to the maze only. Values are shown as relative quantification to handled controls [RQ; RQ_(Control)_ = 1] and mean ± s.e.m. per group. ^*^Significant difference from Control with *p* < 0.05; ^#^significant difference between Spatial Learning and Water Exposure groups with *p* < 0.05.

Exposure to the MWM significantly affected mRNA expression level of selected target genes in the BLA as demonstrated by One-Way ANOVA [Figure 2; *GAD67*: *F*_(2, 22)_ = 7.679, *p* < 0.05; *GAD65*: *F*_(2, 23)_ = 6.204, *p* < 0.05; *NPY*: *F*_(2, 23)_ = 4.113, *p* < 0.05; *SST*: *F*_(2, 23)_ = 3.911, *p* < 0.05]. Further analysis using LSD *post hoc* test revealed lower levels of *GAD65* and *GAD67* expression in the Spatial Learning stress group compared to the other two groups (*p* < 0.05). *SST* and *NPY* mRNA expression levels were reduced as well after Spatial Learning stress, but only when compared to the Water Exposure stress group (*p* < 0.05), which in turn was somewhat increased in expression of both neuropeptides compared to Control. The same effect was observed for *CCK* mRNA expression (non-parametric chi square test: χ^2^ = 8.508, *p* < 0.05).

### Cort plasma levels

**Figure 3:**
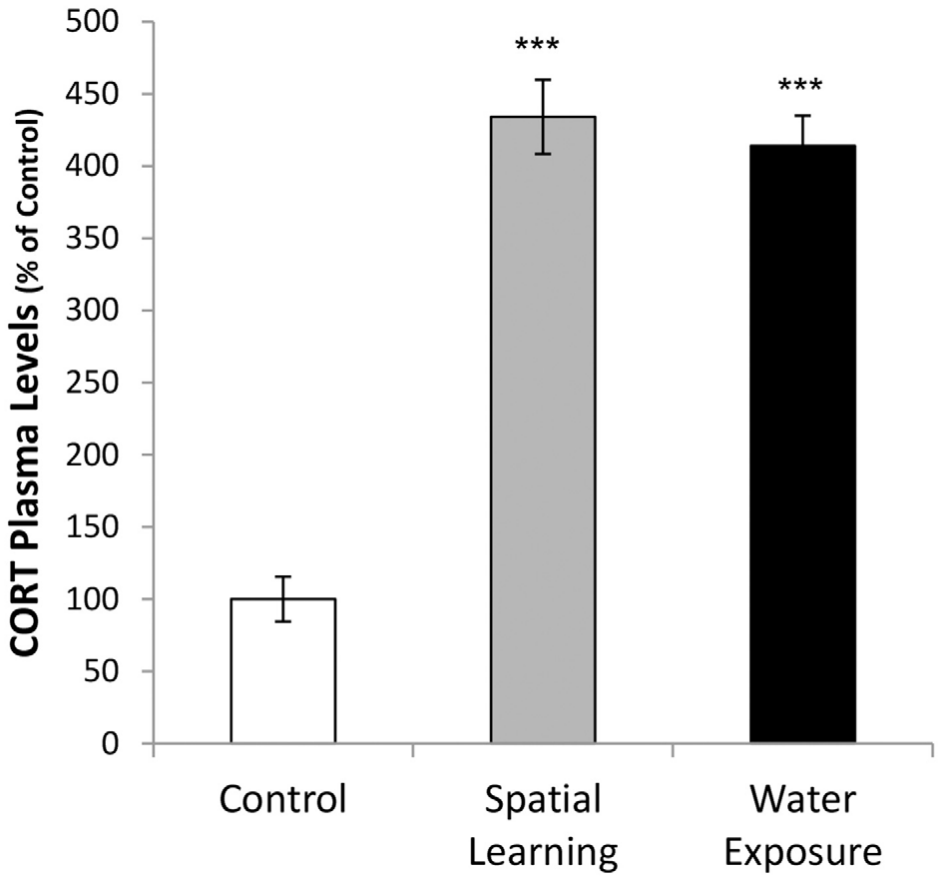
**Activation of hormonal stress response by water maze experience**. Corticosterone (CORT) plasma concentrations were increased after both, water maze exposure alone and with an invisible platform enabling spatial learning, compared to handeled Controls (CORT concentration Control = 386 ± 60 ng/ml). Values are mean ± s.e.m. ^***^Significant difference from Control with *p* < 0.001.

One-Way ANOVA followed by LSD *post-hoc* comparison revealed that exposure to the MWM affected CORT plasma levels, [Figure 3; *F*_(2, 21)_ = 78.483, *p* < 0.001], with both spatial learning and water exposure increasing CORT plasma levels 4-fold compared to Control group (CORT concentration Control = 386 ± 60 ng/ml; *p* < 0.001 to both groups). No difference was observed between the two groups that have been in the water maze.

### BLA activation

**Figure 4:**
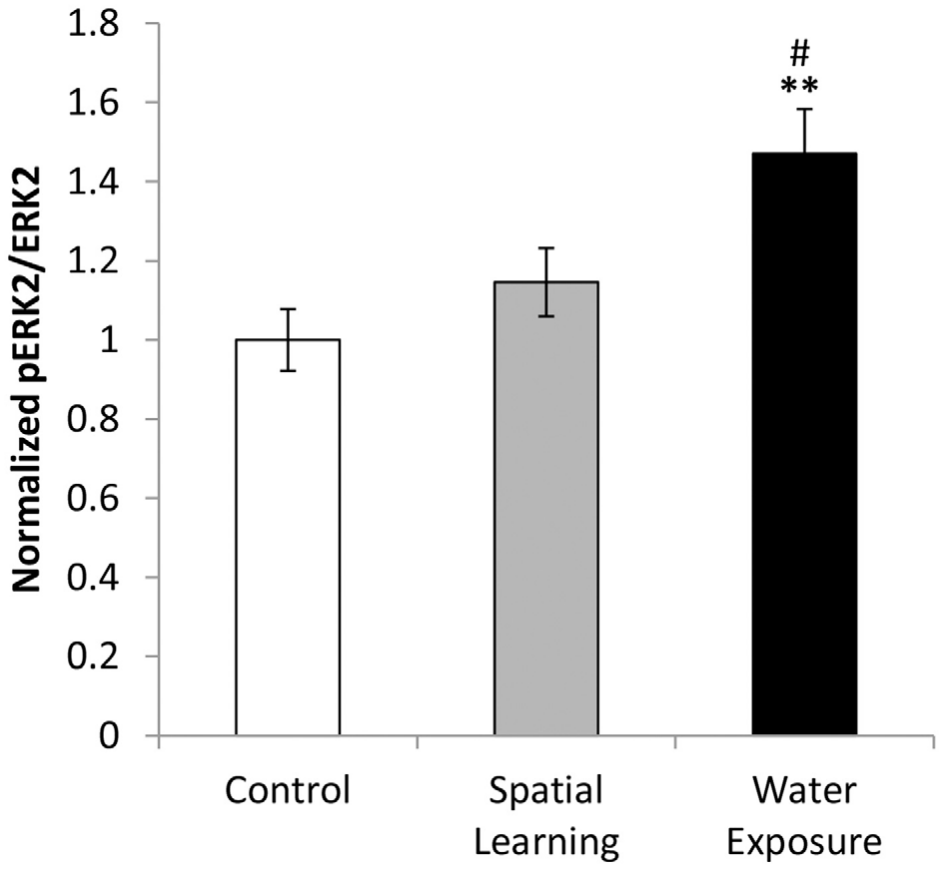
**BLA activation by water exposure stress without the possibility of spatial learning**. While water exposure alone increased activation of the BLA as indicated by enhanced levels of ERK 1/2 phosphorylation, no such effect was observed after by providing an invisible platform enabling for spatial learning. Values are mean ± s.e.m. for normalized optical density (arbitrary units). **Significant difference from Control with *p* < 0.01; ^#^significant difference between Spatial Learning and Water Exposure groups with *p* < 0.05.

The BLA was differentially activated in the different groups as indicated by phosphorylation of ERK 1/2 [Figure 4; One-Way ANOVA: *F*_(2, 21)_ = 6.644, *p* < 0.01]. Further analysis using LSD *post hoc* test revealed increased ERK activation of the BLA after water exposure only (*p* < 0.01 compared to Control and *p* < 0.05 compared to Spatial Learning group), while no difference in ERK 1/2 phosphorylation level was observed between the Control group and the Spatial Learning group.

## Discussion

The MWM task is commonly used for the evaluation of learning and memory in rodents, however the complex involvement of the GABAergic system in these processes has not yet been completely solved (D'Hooge and De Deyn, 2001). Here we demonstrate changes in molecular markers of GABAergic function in subregions of the hippocampus and the BLA after spatial learning in this task. In addition, we show a differential expression of the selected target genes in the BLA after spatial learning compared to exposure to the water maze itself. Moreover, a selective increase in the phosphorylation within the BLA of ERK 1/2 was observed in animals that have been exposed to the water maze without the possibility to learn the location of the escape platform, whereas CORT plasma levels were increased in both compared to handled control animals. These data suggest a differential involvement of hippocampal local GABAergic circuit neurons in spatial learning.

Spatial learning of the platform location induced a distinct pattern of changes in expression of the GABA-synthetizing enzymes *GAD65* and *GAD67* as well as in the expression of selected neuropeptides *NPY*, *SST*, and *CCK* that modulate GABAergic neurotransmission and mark different subsets of GABAergic interneurons. Intriguingly, the pattern of changes was subregion-specific, with increased levels of *NPY* in the DG, increased levels of *SST* in the CA3 and decreased levels of *CCK* in the CA1 region of the dorsal hippocampus. These changes may highlight the activation of distinct subsets of interneurons that are preferential located in a specific subregions of the hippocampus to modulate local network activity (Maccaferri and McBain, 1995; Houser, 2007) thereby shaping the information flow throughout the hippocampal formation.

The DG as the main input station to the hippocampal formation is strongly modulated by its local inhibitory network (Acsady and Kali, 2007); GABAergic interneurons thus contribute to the DG function in pattern separation, with implications for spatial memory formation (Richter-Levin et al., 1995; Acsady and Kali, 2007; Kesner, 2007). An important interneuron subpopulation that exerts feedback inhibition on DG inputs are the so-called HIPP cells (= hilus perforanth path associated cells),that have their cell bodies located in the hilus and send axons to the distal dendrites of DG granule cells (Houser, 2007; Sperk et al., 2007). The increased *NPY* mRNA expression after spatial learning in the MWM, without additional significant changes for *GAD65* and *GAD67* or the other neuropeptides, therefore most likely reflect a training-specific activation of these *NPY*-positive HIPP cells subpopulation. The contribution of *NPY* appears to be region-specific, since a general increase in *NPY* by constitutive overexpression, in mice, impairs spatial memory in the MWM (Thorsell et al., 2000). Indeed, in our experiments we observed no modulation of *NPY* expression in the SO of dorsal hippocampus areas CA1 and CA3 after spatial learning.

However, in the CA3 region we found a learning-induced increase in *SST* expression. *SST* in interplay with serotonergic signaling in the hippocampus is required for spatial memory formation in the MWM (Dyer and Cain, 2007) and depletion of *SST* impairs spatial learning (Matsuoka et al., 1995). The CA3 region of the dorsal hippocampus is one of the key regions for acquisition of spatial memory in the water maze (Florian and Roullet, 2004; Teather et al., 2005) and *SST* can facilitate LTP of mossy fiber inputs into this region, but not into CA1 (Matsuoka et al., 1991). In our study the SO of the CA3/CA1 was isolated via LCM and in this region *SST* is confined to the oriens/lacunosum-moleculare (OLM) cells. Comparable to the HIPP cells in the DG, these cells exert feedback inhibition on principal cells and shape the information flow in the hippocampal formation (Katona et al., 1999). OLM cells further elicit and control rhythmical activity in the hippocampus (Somogyi and Klausberger, 2005) that is essential for spatial and temporal integration during navigation and memory formation (Buzsaki and Moser, 2013).

In contrast to these selective increases of neuropeptide expression in hilus and CA3, changes in the CA1 region were characterized by reduced expression of the GABA synthesizing enzymes *GAD65* and *GAD67*. This indicates a more general activation of local interneurons in CA1 and may potentially support spatial memory formation by reducing inhibition in CA1 pyramidal cells, which are critical for this form of learning (Teather et al., 2005; Goodrich-Hunsaker et al., 2008). In fact, drugs that enhance GABAergic signaling by allosteric modulation of GABA_A_ receptors impair spatial learning in the MWM (White et al., 1997). Finally, the reduction in *CCK* expression observed under spatial learning conditions in the CA1 also supports spatial memory formation. Within the CA1 region, *CCK* is mostly expressed in basket cells, with cell bodies located in the stratum pyramidale. These cells are involved in stress coping and emotional memory formation via inputs from the serotonergic and cannabinoid system (Touma, 2011; Haring et al., 2012; Keimpema et al., 2012) and control rhythmic activity of principal cells. *CCK*-positive interneurons are also observed in the SO (Schiffmann and Vanderhaeghen, 1991; Tsou et al., 1999), but the function of this subpopulation is not well understood.

Together, these data demonstrate that spatial learning in the water maze induces a distinct subregion-specific expression pattern of *GAD65* and *GAD67* as well as of the selected neuropeptides. Exposure to the water maze alone induced no alterations in hippocampal mRNA expression levels for any of the factors examined.

However, in the BLA we observed a differential expression between groups being only exposed to the water maze or gaining spatial memory during the task. Here, *GAD65* and *GAD67* as well as the neuropeptides *NPY*, *SST*, and *CCK* show a rather reduced expression after spatial learning compared to the water exposed animals. For *GAD*65 and *GAD67* reduced expression in the BLA was observed in the spatial learning group compared to handled controls, indicating effects of spatial learning on the amygdala expression of these factors. A learning-specific reduction in *GAD65* mRNA in the BLA has also been reported after fear conditioning (Bergado-Acosta et al., 2008). Such a reduction appears to be linked to transiently reduced inhibition in the BLA (Szinyei et al., 2007) and a reduction in extracellular GABA (Stork et al., 2002) that may contribute to memory consolidation. Indeed, GABAergic signaling in the BLA can modulate spatial memory formation and LTP in the hippocampus (Kim et al., 2005) and both regions, BLA and hippocampus, are activated during retrieval of spatial memory (Vanelzakker et al., 2011).

The differential expression of neuropeptides in the BLA may reflect, as in the hippocampus, the activation of specific interneuron subpopulations that could contribute to altered LTP and inhibitory control observed after stress (Vouimba et al., 2004; Rodriguez Manzanares et al., 2005). On the other hand, *NPY* and *SST* themselves are powerful modulators of anxiety-related behavior and have anxiolytic properties when administered to the amygdala (Heilig, 2004; Yeung and Treit, 2012). Increased levels of *NPY* in the amygdala have been linked to coping behavior after repeated exposure to stressors (Thorsell et al., 1999) and in an animal model of posttraumatic stress disorder, increased levels of *NPY* in amygdala and hippocampus have been associated with resilience to traumatic stress (Cohen et al., 2012). *SST* microinfusions in the amygdala produce anxiolytic- and antidepressant-like effects (Yeung and Treit, 2012), likely through modulation of GABA_A_ receptor signaling (Engin et al., 2008), whereas administration of *CCK* elicits anxiety-like behavioral responses (Mathew et al., 2008; Sherrin et al., 2009). Thus the transcriptional regulation of these factors in the BLA likely is part of an adaptive response to a stressful experience.

Analysis of CORT plasma levels revealed increases in both, the Spatial Learning and the Water Exposure groups, compared to Control. Elevated CORT reflects the activation of the hypothalamus-pituitary-adrenocortical axis (Armario et al., 2012) and is an important marker for the hormonal stress response. Stress itself thereby consists of different components and includes in part stress by physical activity during swimming (Zheng et al., 2006), but also psychological components due to exposure to a new, challenging environment (the water at a certain temperature; Akirav et al., 2001).

Stress further increases the expression of plasticity markers in the BLA (Zoladz et al., 2012) and activates the signaling and transcription factor ERK 1/2 in this region through phosphorylation (Maldonado et al., 2013). Strikingly, only animals that had been exposed to the water maze without an invisible platform displayed an increase in p-ERK 1/2 in the BLA. However, the increase in p-ERK 1/2 levels in the water exposed animals can neither be attributed to spatial learning per se nor to stress per se, since similar CORT levels were observed in both experimental groups. Consequently, the observed increase in ERK 1/2 phosphorylation may be related to another main difference between the experimental groups: the level of gained controllability over a stressful situation.

While animals that were allowed to learn the location of an invisible platform can be said to have gained controllability over the stressful exposure to the water, animals that have been exposed to the water for the same duration (exposure time was matched to average of learning group per trial) but without an escape platform were left in an uncontrollable situation. Controllability thereby describes the perceived ability to alter the onset, duration, intensity or pattern of an aversive experience (Maier and Seligman, 1976) and contributes strongly as a psychological factor to the emotional impact of a stressful event. The lack of controllability can interfere with the performance in cognitive tasks (Overmier and Seligman, 1967; Seligman and Maier, 1967). In turn, by acquiring escape strategies subjects may effectively reduce the stress levels associated with a given task. We have previously shown that gaining controllability over a stressful situation, i.e., by avoidance learning in a two-way shuttle box, attenuates phosphorylation of ERK 1/2 in the amygdala (Ilin and Richter-Levin, 2009).

Therefore, it is interesting to compare the observed effects between the experimental groups in the light of controllability as a factor. Although their physical stress experience, i.e., the exposure to the water and the swimming itself, was matched, differential expression profiles and transcriptional activation of the BLA were observed between the Water Exposed and the Spatial Learning groups. Animals in both groups may share the motivation to end the challenging and stressful situation of the water exposure, but only the Spatial Learning group can actively reach this goal by learning the location of the hidden platform. Thus, spatial learning in the MWM may be described as leading to gaining controllability over a stressful situation. In line with that, the differential changes in the BLA observed in this study might be, at least in part, related to the difference in the level of gained controllability. Since the BLA critically affects information processing in the hippocampus and hippocampus-dependent memory (Kim et al., 2005) in a subregion-specific manner (Vouimba and Richter-Levin, 2005), the observed changes could contribute also to the differential effects of water exposure on LTP in the DG versus the CA1 reported previously (Kavushansky et al., 2006).

In summary, in this study we could demonstrate a highly specific expression regulation of the interneuron markers *NPY*, *SST*, and *CCK* in distinct subregions of the dorsal hippocampus by spatial learning in the MWM. In the BLA, while spatial learning itself led to reduced expression of *GAD65/67*, a differential expression of all selected markers was observed in water exposed animals compared to animals that learned the location of a hidden escape platform. Together with the increased ERK 1/2 phosphorylation after water exposure only, the observed changes in the BLA appear to be related to the stress experience and may related also to aspects of lack of controllability. Future studies will address how these molecular changes affect information processing in the amygdalo-hippocampal system and the adaptation to learning under stress.

## Author contributions

Osnat Hadad-Ophir, Anne Albrecht, Oliver Stork and Gal Richter-Levin conceived and designed the experiments. Osnat Hadad-Ophir performed the experiments. Osnat Hadad-Ophir, Anne Albrecht, Oliver Stork and Gal Richter-Levin analyzed and discussed the data. Osnat Hadad-Ophir, Anne Albrecht, Oliver Stork and Gal Richter-Levin wrote the manuscript.

### Conflict of interest statement

The authors declare that the research was conducted in the absence of any commercial or financial relationships that could be construed as a potential conflict of interest.

## Supplementary material

Figure S1 **“Invisible platform” learning curve**. Adult male rats (*N* = 10) underwent spatial learning in the Morris water maze for locating a hidden underwater platform in 12 trials (~60 min) with intertrial intervals of 4 min. In all trials rats were allowed to search for the hidden platform for 60 s at maximum with time measured until animal has reached the platform (escape latency). If an animal failed to reach the platform after 60 s, it was placed and left there by the experimenter for 15–30 s. The escape latency was assessed for each trial in order to gaining a learning curve for the controllable stress group. Animals of the uncontrollable stress group were exposed to the same amount of time in each trial as the controllable stress group.
