# Supplementary material for: Amygdala activation and GABAergic gene expression in hippocampal sub-regions at the interplay of stress and spatial learning
Source: Front Behav Neurosci. 2014 Jan 21;8:3. doi: 10.3389/fnbeh.2014.00003 (PMC3896990; doi:10.3389/fnbeh.2014.00003)
Supplement: Figure S1 — “Invisible platform” learning curve. Adult male rats (N = 10) underwent spatial learning in the Morris water maze for locating a hidden underwater platform in 12 trials (~60 min) with intertrial intervals of 4 min. In all trials rats were allowed to search for the hidden platform for 60 s at maximum with time measured until animal has reached the platform (escape latency). If an animal failed to reach the platform after 60 s, it was placed and left there by the experimenter for 15–30 s. The escape latency was assessed for each trial in order to gaining a learning curve for the controllable stress group. Animals of the uncontrollable stress group were exposed to the same amount of time in each trial as the controllable stress group. [file DataSheet1.DOCX]

**Supplemental material**

**Figure S1: 'Invisible platform' learning curve.** Adult male rats (N=10) underwent spatial learning in the Morris water maze for locating a hidden underwater platform in 12 trials (~60 min) with intertrial intervals of 4min. In all trials rats were allowed to search for the hidden platform for 60s at maximum with time measured until animal has reached the platform (escape latency). If an animal failed to reach the platform after 60s, it was placed and left there by the experimenter for 15-30s. The escape latency was assessed for each trial in order to gaining a learning curve for the controllable stress group. Animals of the uncontrollable stress group were exposed to the same amount of time in each trial as the controllable stress group.
